# Impact of Rootstock and Season on Red Blotch Disease Expression in Cabernet Sauvignon (*V. vinifera*)

**DOI:** 10.3390/plants10081583

**Published:** 2021-07-31

**Authors:** Arran C. Rumbaugh, Raul C. Girardello, Monica L. Cooper, Cassandra Plank, S. Kaan Kurtural, Anita Oberholster

**Affiliations:** 1 Department of Viticulture and Enology, University of California, Davis, One Shields Avenue, Davis, CA 95616, USA; acrumbaugh@ucdavis.edu (A.C.R.); rgigardello@ucdavis.edu (R.C.G.); cmplank@ucdavis.edu (C.P.); skkurtural@ucdavis.edu (S.K.K.); 2 University of California Cooperative Extension, 1710 Soscol Avenue, Napa, CA 94559, USA; mlycooper@ucanr.edu

**Keywords:** grapevine red blotch virus, grape ripening, disease expression, season, rootstock

## Abstract

Grapevine red blotch virus (GRBV), the causative agent of grapevine red blotch disease, is widespread across the United States and causes a delay in ripening events in grapes. This study evaluates the effects of GRBV on Cabernet Sauvignon grape berry composition, grafted on two different rootstocks (110R and 420A) in two seasons (2016 and 2017). Total soluble solids, acidity, and anthocyanin concentrations were monitored through ripening and at harvest. Phenolic and volatile compounds were also analyzed at harvest to determine genotypic and environmental influences on disease outcome. Sugar accumulation through ripening was lower in diseased fruit (RB (+)) than healthy fruit across rootstock and season. GRBV impact was larger in 2016 than 2017, indicating a seasonal effect on disease expression. In general, anthocyanin levels and volatile compound accumulation was lower in RB (+) fruit than healthy fruit. Total phenolic composition and tannin content was higher in RB (+) fruit than healthy fruit in only 110R rootstock. Overall, GRBV impacted Cabernet Sauvignon grape composition crafted on rootstock 110R more than those crafted on rootstock 420A.

## 1. Introduction

Grapevines are susceptible to the highest number of pathogens to infect a single crop, with over 70 viruses detected [1]. In 2008, a new virus was first observed in Napa County, California, which economically threatened grapevines: grapevine red blotch virus (GRBV) [2]. This virus is the causative agent of grapevine red blotch disease (GRBD) [3], which has been identified in vineyards across the United States, Canada, Argentina, Mexico, South Korea, and India [4,5,6,7,8,9]. Reports indicate GRBV primarily spreads through propagation material and secondarily through an insect vector [10,11]. *Spissistilus festinus* (Membracidae) was shown to successfully transmit GRBV in greenhouse settings, yet this has not been replicated in vineyards to date [12]. GRBV has been identified as a virus from the *Geminiviridae* family containing a circular single-stranded DNA genome [13,14] similar to other geminiviruses [15]. GRBD expresses symptoms of reddening of leaf blades and margins, with reddening of the primary, secondary, and tertiary veins in red grape cultivars [10]. 

GRBV affects grapevines in various ways. For example, leaves on infected vines show increased levels of sugar, phenolics, particular amino acids, and enzymatic activity related to plant defense, as well as a reduction in carbon fixation [16,17,18]. However, the most damaging are the effects on grape composition [17,18,19,20] which has been shown to be detrimental to final wine quality [21]. GRBV delays ripening by decreasing the accumulation of sugar and anthocyanin in berries, potentially due to the impairment of translocation mechanisms [17,18,20]. The virus has variable impacts on primary and secondary metabolites, specifically phenolic and aroma compounds [17,19,20]. In summary, detrimental economic impacts to vineyards in the United States could reach $68,548/ha with vine removal being the only current method of alleviation [22]. Consequently, recent research has strived to understand the effects and functioning of GRBV to establish mitigation strategies to alleviate the impact on grape composition and wine quality.

The grape berry has a double sigmoidal growth curve with three distinct phases. The first phase is characterized by cell division and production of seeds, as well as synthesis of tannins and organic acids. The second phase is characterized with the onset of veraison, which is when the grape berry begins to soften and change color. The final and third phase is berry engustment/ripening, where berries increase in size, sugar accumulates, acidity declines, and secondary metabolites such as anthocyanins and aromatic compounds are synthesized inside the berry [23]. Studies have shown that volatile compounds such as terpenoids and C6 compounds, begin to accumulate in berries after veraison, and are controlled by numerous factors [24,25,26,27,28]. The synthesis of these compounds in berries is also altered by external factors such as light exposure or pathogens [29,30]. In addition, these secondary metabolites are crucial to grape growers and winemakers due to their importance in the quality of a final wine [31]. Grape maturity has shown to be a key driver in the composition of a final wine, where later harvested fruit produces wines with lower concentrations of C6 alcohols (vegetal aromas) and higher in concentration of esters (fruity aromas) [32]. However, the impacts of GRBV on volatile compound abundance in harvested grapes has not been investigated.

A plant’s genetic material may influence susceptibility to viral infections [33,34,35]. Additionally, rootstocks can impact grapevine physiology and impact the overall composition of a grape berry. For instance, rootstock 110R (*Vitis berlandieri × Vitis rupestris*) causes high vigor and high drought tolerance in grapevines; whereas 420A (*V. berlandieri × Vitis riparia*) is a rootstock of low to moderate vigor and low drought resistance [36]. Vigor, resulting in greater shoot length and hence leaf area, may impact net carbon assimilation and the translocation of metabolites into the berry, consequently affecting the final wine composition [37]. These hydric differences affecting carbon metabolism in rootstocks can also impact the plant-pathogen interactions. Therefore, it is plausible that severity of GRBD symptoms will be dependent on the interaction between scion cultivar-rootstock. However, this has not been fully investigated. Macro and micro climate fluctuations may also be a factor in pathogen-plant interactions [38,39], and should be considered.

This study investigated the impact of GRBV on the biosynthesis and accumulation of primary and secondary metabolites in grape berries throughout ripening and at harvest. Additionally, the influence of seasonal and genotypic factors on disease expression within grapevines were studied.

## 2. Results

### 2.1. GRBV Impacts on Grape Maturation

**Figure 1:**
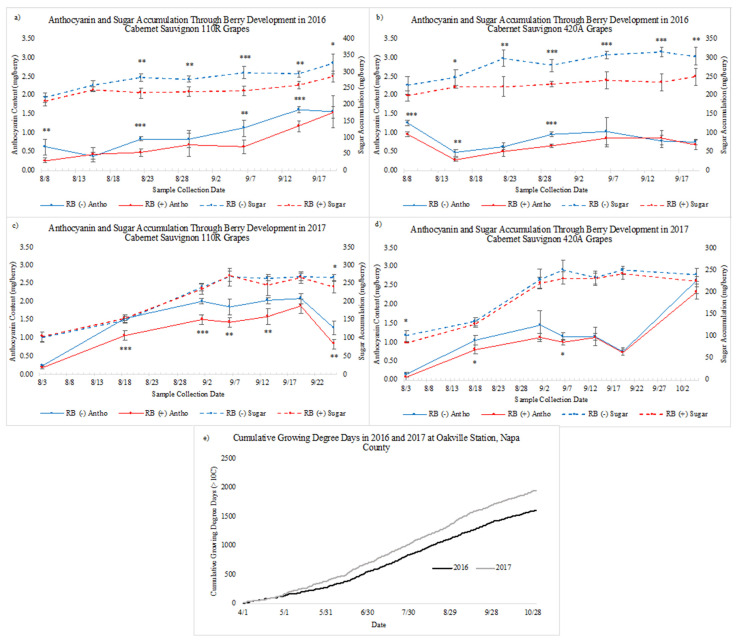
Sugar accumulation and anthocyanin content through ripening from pre-veraison to harvest for (**a**) sugar accumulation in 2016 (**b**) anthocyanin content in 2016 (**c**) sugar accumulation in 2017, (**d**) anthocyanin content in 2017 (*n* = 5), (**e**) cumulative growing degree days (>10 °C). CS = Cabernet Sauvignon, RB = red blotch, (−) = negative, and (+) = positive. Asterisks indicate a significant difference between RB (−) and RB (+) after an ANOVA (* = *p* < 0.05, ** = *p* < 0.01, *** = *p* < 0.001).

**Figure 2:**
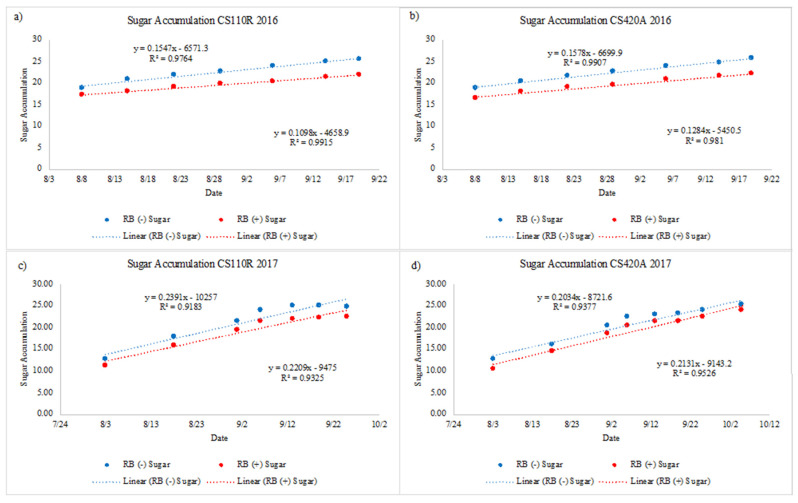
The rate of sugar accumulation as °Brix through ripening for RB (−) and RB (+) data vines. (**a**) CS 110R in 2016, (**b**) CS 420A in 2016, (**c**) CS 110R in 2017, and (**d**) CS 420A in 2017 (*n* = 5). TA = Titratable Acidity, CS = Cabernet Sauvignon, RB = red blotch, (−) = negative, and (+) = positive.

**Figure 3:**
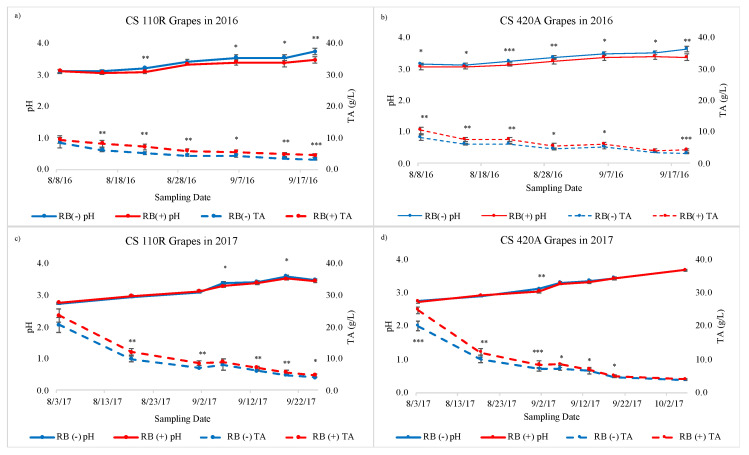
Titratable acidity and pH values from pre-veraison to harvest for (**a**) CS 110R in 2016, (**b**) CS 420A in 2016, (**c**) CS 110R in 2017, and (**d**) CS 420A in 2017 (*n* = 5). TA = Titratable Acidity, CS = Cabernet Sauvignon, RB = red blotch, (−) = negative, and (+) = positive. Asterisks indicate a significant difference between RB (−) and RB (+) after an ANOVA (* = *p* < 0.05, ** = *p* < 0.01, *** = *p* < 0.001).

Figure 1, Figure 2 and Figure 3 depict sugar accumulation, anthocyanin levels, TA, and pH through ripening. Sugar accumulation was determined by converting °Brix to mg of sugar per berry [40]. Anthocyanin content was lower in RB. (+) grapes when compared to RB (−) grapes for both years and rootstocks during ripening (Figure 1). However, the degree of impact varied depending on season and rootstock. In 2016, both rootstocks were equally impacted throughout ripening regarding sugar accumulation and anthocyanins levels. However, in 2017 sugar accumulation was generally not significantly impacted by disease status. In 2017, grape anthocyanin levels were more significantly impacted for infected vines on 110R rootstock than 420A rootstock, whereas rootstock impact was less apparent in 2016. At harvest in 2016 (20 September), CS 110R and 420A rootstocks, respectively, had a 2% and 11% decrease in anthocyanin content (mg/berry) and a 12% and 18% decrease in sugar content (mg/berry) in RB (+) grapes when compared to RB (−) grapes. In 2017, at harvest (26 September and 6 October), anthocyanin content was 35% and 11% lower in RB (+) when compared to RB (−), and sugar content was 9% and 7% lower, for 110R and 420A, respectively.

By plotting °Brix over ripening and fitting a linear trendline, it is possible to compare the rate of ripening for RB (−) and RB (+) grapevines (Figure 2). As indicated by the slope of the best fit line, the rate of ripening was always higher for RB (−) data vines when compared to RB (+) data vines, with the exception for CS 420A in 2017. In addition, the rate was also lower in 2016 than 2017 across virus status and rootstocks. Interestingly, the difference in the rate of ripening between RB (−) and RB (+) data vines was larger in 2016 than in 2017 which correlates to the larger differences in accumulated sugar at harvest. In 2017, the rate was lower for CS 420A than CS 110R across virus status.

Differences between RB (−) and RB (+) for pH and TA also varied between years and rootstocks (Figure 3). In general, RB (+) grapes had lower pH values and a higher TA when compared to RB (−), which agrees with results found by Martínez-Lüscher et al. [17]. For both TA and pH, there were more sampling dates significantly different between RB (+) and RB (−) observed in 2016 than in 2017, similar to sugar accumulation (Figure 1). 

### 2.2. GRBV Impacts on Grape Composition at Harvest

In general, there were no significant differences in yield and cluster number between RB (+) and RB (−) grapevines, except for CS 110R in 2017. In latter case, the yield and number of clusters per vine were significantly higher in RB (+) than RB (−) (Table 1), contrary to findings by Martínez-Lüscher et al. [17], in which a smaller subset of data vines was used for yield components, potentially explaining the variation. As with previous findings, GRBD consistently decreased °Brix and pH values, while increasing TA values in grapes at harvest, indicating GRBD causes a delay in ripening [2,13,17,18,20,41]. In addition, malic acid concentrations in RB (−) grapes were in general significantly lower than RB (+) grapes.

There was a significant effect from virus status on °Brix, pH, TA, yield, and clusters/vine values. In addition, there was a significant interaction for virus status to year for °Brix, pH and TA values, indicating that environmental factors play a role in disease expression for these parameters. For these values, Table 1 shows that grapevines in 2017 were less impacted by GRBD than in 2016, as seen during grape ripening (Figure 1 and Figure 2). 

### 2.3. Grape phenolic Profile

**Figure 4:**
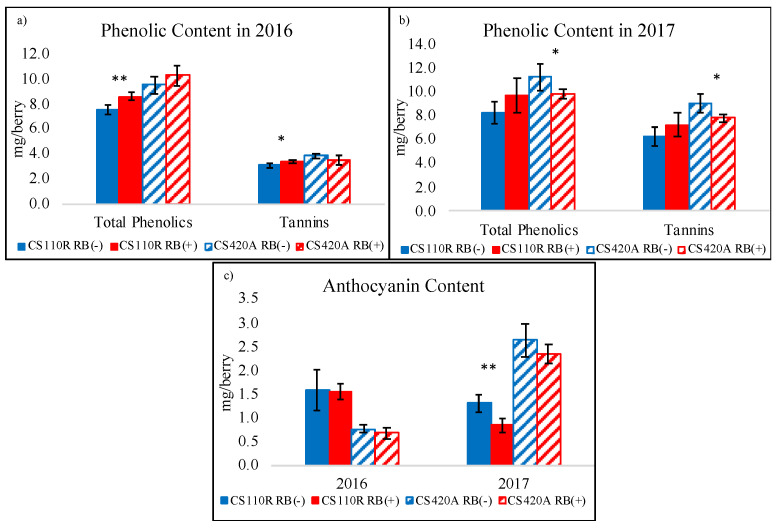
Phenolic profile of whole berry extracts at harvest through protein precipitation analysis. (**a**) Total phenolic and total tannin composition from CS grapes on 110R and 420A rootstock in 2016, (**b**) total phenolic and total tannin composition from CS grapes on 110R and 420A rootstock in 2017, and (**c**) total anthocyanin concentrations in CS grapes in 2016 and 2017 (*n* = 5). CS = Cabernet Sauvignon, RB = red blotch, (−) = negative, and (+) = positive. Asterisks indicate a significant difference between RB (−) and RB (+) after an ANOVA (* = *p* < 0.05, ** = *p* < 0.01, *** = *p* < 0.001).

The grape phenolic profile in 2016 and 2017 from the protein precipitation assay is shown in Figure 4. It should be noted that the phenolic content is expressed in mg/berry to observe differences in biosynthesis in the berries. Similar trends as in Figure 4 were observed for phenolic concentrations (mg/g of berry, Table S1).

In general, it was observed that RB (−) grapes were higher in anthocyanin levels than RB (+) grapes, which was only significant in 2017 for CS 110R. For CS 110R grapes, total phenolic and total tannin concentrations in RB (+) grapes were higher than in RB (−) grapes. Larger differences between RB (−) and RB (+) in overall phenolic content was observed in 2017 when compared to 2016; however, these differences were not always significant.

Content (mg/berry) and concentration (mg/ g of berry) of total phenolics, tannins, and anthocyanins were analyzed through a three-way ANOVA with three-way interactions (Table S1). Results indicate that there was a significant virus status and virus status to year interaction for anthocyanin concentrations and content. This suggests that there was a larger interaction between disease status and season, rather than the rootstock, on final anthocyanin content in grapes. In addition, there was a significant virus status effect and virus status to rootstock effect for total tannins and total phenolics, indicating that the disease outcome is also a factor of rootstock for these parameters. 

### 2.4. Volatile Analysis- HS-SPME-GC-MS

**Figure 5:**
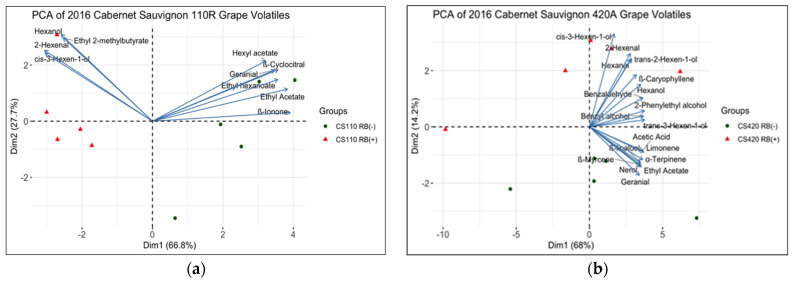
Principal component analysis of significantly different volatile compounds in whole berry extracts from CS grapes on (**a**) 110R and (**b**) 420A rootstock from 2016 (*n* = 5). CS = Cabernet Sauvignon, RB = red blotch, (−) = negative, and (+) = positive.

**Figure 6:**
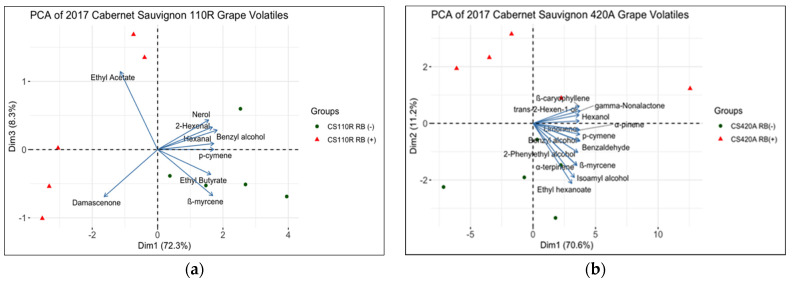
Principal component analysis of significantly different volatile compounds in whole berry extracts from CS grapes on (**a**) 110R and (**b**) 420A rootstock from 2017 (*n* = 5). CS = Cabernet Sauvignon, RB = red blotch, (−) = negative, and (+) = positive.

The volatile compound profiles of RB (−) and RB (+) grapes were determined in both 2016 and 2017 seasons (Table S2). PCA was performed to plot the variability between RB (−) and RB (+) grape samples (Figure 5 and Figure 6). Between 80.6- 94.5% of the variance is explained by the PCA in Figure 4 and Figure 5. For CS 110R, only the significantly different volatile compounds between RB (−) and RB (+) are plotted. There were, respectively ten and nine significant volatile compounds that explained the difference between treatments for CS 110R in 2016 and 2017. For CS 420A, the volatile compounds that contributed most to the variance of the PCA were plotted, due to few volatile compounds being significantly different. This selection was based on the squared cosine (cos^2^=0.90) which shows the importance of the volatile compounds to explain the variance in the data [42]. For CS 420A, in 2016, only cis-3-hexen-1-ol was significantly different, and in 2017, only β-linalool and β-citronellol were significantly different with a α level of 0.95.

In 2016, it was observed that cis-3-hexen-1-ol, hexanol, 2-hexenal, ethyl-2-methylbutyrate, and trans-2-hexen-1-ol were highly correlated with RB (+) grape extracts in CS 110R and CS 420A (Figure 5), whereas the volatile compounds hexyl acetate, ethyl acetate, ethyl hexanoate, geranial, ß-ionone, and ß-cyclocitral were correlated with CS 110R RB (−) grapes (Figure 5a). As for CS 420A, in 2016 (Figure 5b), limonene, ß-linalool, ß-myrcene, acetic acid, α-terpinene, geranial, nerol, and ethyl acetate were correlated to RB (−).

The volatile profile of grapes in 2017 was similar to those in 2016. However, 110R RB (+) grapes were correlated to ethyl acetate and ß-damascenone (Figure 6a). On the other hand, p-cymene, ethyl butyrate, ß-myrcene, benzyl alcohol, 2-hexenal, hexanal, and nerol were correlated with CS 110R RB (−) grapes. Figure 6b indicates that CS 420A RB (+) grapes were only correlated with γ-nonalactone, ß-caryophllene, and trans-2-hexen-1-ol, whereas, CS 420A RB (−) grapes were correlated with ethyl hexanoate, isoamyl alcohol, α-terpinene, α-pinene, p-cymene, limonene, benzyl alcohol, benzaldehyde, 2-phenylethyl alcohol, and ß-myrcene. In both years, in general it was observed that RB (+) grapes were correlated with fewer volatile compounds, apart from C6 aldehydes and alcohols.

Lastly, results from the three-way ANOVA with three-way interactions are shown in Table S2. It was observed that ethyl acetate, limonene, 2-hexenal, ethyl hexanoate, p-cymene, hexyl acetate, octanal, trans-2-hexen-1-ol, and ß-ionone had a significant virus status effect across years and rootstocks. Ethyl acetate, 2-hexenal, ethyl hexanoate, hexyl acetate, hexanol, trans-3-hexen-ol, cis-3-hexen-1-ol, trans-2-hexen-1-ol, geranial, ß-damascenone, and ethyl cinnamate had a significant virus status to year interaction, whereas, limonene, 2-hexenal, ethyl hexanoate, p-cymene, cis-3-hexen-1-ol, trans-2-hexen-1-ol, nerol oxide, benzaldehyde, geranial, and benzyl alcohol had a significant virus status to rootstock effect. This suggests that the extent to which these compounds are impacted due to GRBV will vary depending on the season and the genotype of the grapevine. 

## 3. Discussion

### 3.1. Impact on Grape Volatile Compounds

After *veraison*, volatile compound accumulation begins in grapes and changes through ripening [25,43,44]. However, the impacts of GRBV on grape volatile compounds have not been investigated. We found 35 different aromatic compounds in grapes from the two rootstocks over two seasons, of which 24 were similar between the two years studied (Table S2).

In 2016, across rootstock, RB (+) grapes were generally lower in volatile compound levels than RB (−), except for C6 compounds such as 2-hexenal, hexanal, cis-3-hexen-1-ol, trans-2-hexen-1-ol, and hexanol (Figure 5). These C6 volatile compounds are synthesized in the grape skin through the lipoxygenase pathway, are generally responsible for green or grassy aromas [24] and accumulate in CS grapes up to 18 ºBrix [25]. With the exception of hexanol, the levels of these compounds begin to significantly decrease thereafter, with a 67% decrease in grapes at 25 ºBrix when compared to grapes at 18 ºBrix [25]. These observations correlate with the common finding that GRBV causes a delay in ripening [2,10,17,18], with green aromas being present and correlated with the lower sugar accumulation [25]. On the other hand, RB (−) grapes were highly correlated with monoterpenes such as limonene, ß-myrcene, α-terpinene, geranial and p-cymene (Figure 4 and Figure 5), which are responsible for floral and fruity aromas. These compounds have been associated with CS grapes at harvest and are known to increase through grape ripening [26] and may decrease at over ripeness [45,46,47]. 

In addition, RB (−) grapes were also highly correlated with esters such as ethyl acetate, ethyl hexanoate, hexyl acetate, and ethyl butyrate. Although ester formation is mainly related to yeast or bacteria metabolism during winemaking [48,49], grapes are also known to synthesize esters. Anthraniloyl-coenzyme A (CoA):methanol acyltransferase (AMAT) is known to be responsible for the formation of methyl anthranilate in grapes and it is also classified as an ester-forming acyltransferase, which could be responsible for the formation of esters in grapes [50]. The esters found in the current work that in general related mostly to RB (−) grapes, are known to produce red and black fruit aromas [51,52,53]. Collectively, these results confirm that RB (−) grapes underwent normal ripening processes [25] and produced more fruity aromas, while RB (+) grapes at harvest have aroma characteristics more related to early ripening stages.

### 3.2. Impact of Season on Disease Expression

Results indicated that in 2016 GRBD had a larger impact regarding sugar accumulation, pH, TA, and final sugar content (°Brix) than in 2017. In addition, the harvest dates were two to three weeks later in 2017 than in 2016. These observations can potentially be explained by the difference in temperature between the two seasons. In 2017, Napa County experienced a heat wave from 26 August–11 September, where nine days were over 35 °C, and four days were over 40 °C. The cumulative growing degree days for both years can be seen in Figure 1e. 

Extreme heat conditions (>30 °C) during grape maturation have been shown to inhibit enzymatic activity and halt the biosynthesis of metabolites inside the grape berry [54,55,56,57]. Inhibition of these processes due to heat leads to decreases in sugar accumulation and increases in acidity in healthy fruit [58]. This is thought to be caused from a decrease in rate of translocation of sugars from leaves to fruit, through the reduction in photosynthesis at temperatures greater than 30 °C [59]. The rate of ripening in 2017 was faster than 2016 prior to the heat spike (Figure 1 and Figure 2). However, during the heat spike in late August to harvest, sugar accumulation plateaued resulting in extended harvest times in 2017. 

In addition, research has shown that temperature can alter virus-induced gene silencing (VIGS) which is triggered with the infection of a virus as a plant-derived defense mechanism to downregulate the genes of interest [60]. Previous work on other plant species infected with a geminivirus [38,60,61] has shown that the extent of gene silencing is related to temperature. Specifically, Chellappan et al. [38] showed that temperatures over 30 °C induced gene silencing, which interfere with gene expression, resulting in decreases in viral DNA accumulation and decreases in symptoms. Similarly, Flores et al. [60] observed that temperatures above 22 °C attenuated infection symptoms and increased gene silencing. Thus, in 2017, the infected grapevines on both rootstocks could have experienced a reduction in GRBV impacts due to the high temperatures causing viral gene silencing and a decrease in viral DNA. However, the gene expression and regulation of transcriptional factors need to be investigated further to understand the correlation between extreme heat and disease expression in GRBD infected grapevines.

At harvest, a three-way ANOVA indicated that seasonal differences play a large role in the extent of disease symptoms in terms of anthocyanin content at harvest and through ripening (for CS 110R) which was not observed for total tannin and total phenolic content. Past studies have indicated that anthocyanin accumulation in grapes are highly susceptible to variations in temperature, with high temperatures leading to anthocyanin degradation and inhibition of biosynthetic pathways [56,62], whereas tannin concentrations are less sensitive to environmental factors [63,64,65,66]. Therefore, regarding anthocyanin content, the temperature differences between the two seasons may have had a compounding effect with GRBD infection in grapevines.

### 3.3. Differences in Disease Expression Due to Rootstock

Similar to previous results [20], the severity of GRBD symptoms depends not only on season, but also on rootstock. Anthocyanin levels through ripening and at harvest in 2017 for CS 110R infected grapevines were more impacted than in 2016 which was not observed for CS 420A (Figure 1). Previous work described the impact GRBV has on grape metabolism and demonstrated that GRBV inhibits the phenylpropanoid pathway in grapes, which is responsible for the synthesis of flavonoids [41]. As previously mentioned, temperature plays a large role in anthocyanin content in grapes, where higher temperatures lead to lower anthocyanin levels [56,62]. Therefore, it is possible that the extreme heat in 2017 acted as a secondary stressor to infected grapevines, and potentially caused larger decreases in anthocyanin levels through ripening than in 2016. However, this was only observed for rootstock 110R, suggesting that infected grapevines on this rootstock are potentially more susceptible to temperature fluctuations. In addition, the difference in the rate of ripening between RB (−) and RB (+) data vines (Figure 2), was larger for CS 110R than for CS 420A. This indicates that the virus differentially impacted the rate of translocation of sugars from the leaves to the berries depending on the rootstock. 

Additionally, at harvest CS 110R RB (+) grapevines consistently had higher levels of total tannins and phenolics than RB (−) grapes, where the opposite was observed for CS 420A (Figure 4). The former has been seen in prior research by Girardello et al. [20] which screened the impact of GRBD on three varieties across seven sites. One of the varieties which had significantly higher proanthocyanidin (condensed tannins) values in RB (+) grapes compared to RB (−) was CS on rootstock 110R. Flavonoid biosynthesis such as flavan-3-ols and tannins has been correlated to abiotic and biotic stress responses in the grape [67]. It is possible that the higher content of tannin observed in CS 110R infected grapes is correlated to a plant induced defense response, which was less significant in CS 420A. Lastly, the volatile aroma profiles between RB (+) and RB (−) were more similar for grapes from rootstock CS 420A compared to rootstock CS 110R, indicating that choice of rootstock has an influence on disease expression and may have various effects on secondary metabolites.

Plant-pathogen interactions can vary depending on the genetic makeup of the plant [33,34,35]. Rootstock 110R has high drought tolerance and is a moderately high vigor rootstock; whereas 420A has less drought tolerant and induces lower vigor in the scion in comparison. Lower vigor can result in a change in microclimate by increasing sun exposure, overall changing berry ripening and composition [62,63,64,68]. Previous research that investigated the impact of GRBV on vine physiological found similarly that CS110R grapes exhibited more symptoms than CS 420A [17]. In this study, RB (+) grapevines had higher sugar content in the leaves, lower sugar content in the grapes, and higher water potential than RB (−) grapevines. These differences were more drastic for CS 110R than CS 420A grapevines. In addition, CS 110R had higher water potential than CS 420A across disease status, correlating to the high vigor of 110R. Overall, this study concluded that GRBV inhibited the translocation mechanisms of photosynthetic products from the source (leaves) to the sink (grapes). Taken together, this suggests that there is a larger impairment to translocation mechanisms in the CS 110R grapevine than CS 420A grapevines. 

## 4. Materials and Methods

### 4.1. Chemicals and Reagents

All water used during extractions and other analyses was 18MΩ·cm deionized water from a Milli-Q Element system (Millipore, Bedford, MA, USA). All ethanol was purchased from KOPTEC (Decon Labs, King of Prussia, PA, USA). ACS grade acetone was used during phenolic extractions, along with 37% HCl, which was purchased from Sigma Aldrich (St. Louis, MO, USA). Ascorbic acid, maleic acid, bovine serum albumin, glacial acetic acid, ferric chloride, triethanolamine, and NaCl were purchased from Sigma Aldrich (St. Louis, MO, USA). Urea and NaOH were purchased from Thermo Fischer (Waltham, MA, USA), and potassium bitartrate and potassium metabisulfite were purchased from ACROS organics-Thermo Fischer (Fair Lawn, N, USAJ). For headspace solid-phase microextraction-gas chromatography-mass spectrometry (HS-SPME-GC-MS) analysis, sodium citrate dehydrate was purchased from Thermo Fischer (Waltham, MA, USA). Internal standards, 2-octanol and 2-undecanone were purchased from Sigma Aldrich (St. Louis, MO, USA).

### 4.2. Plant Material

We used Cabernet Sauvignon grapevines (clone 8, Foundation Plant Services, University of California, Davis) grafted onto 110R and 420Vineyard (Napa County, CA, USA). The grapevines were trained to a bilateral cordon, in a vertical shoot positioned system. Vineyard management followed standard commercial practices for the region. The grapevines were drip-irrigated at 50% of crop evapotranspiration as reported previously [17]. For several years prior to the initiation of this study, GRBD symptoms had been monitored for each vine in this block. Petiole samples from a subset of vines from this block were tested by qPCR analysis at Agri-Analysis LLC laboratories in Davis, CA to confirm the healthy and GRBV status of the grapevines [12]. In addition, the plant material was screened for the presence of the three most common grapevine leafroll associated virus (GLRaV-1, 3, and 4) as well as Rupestris stem pitting-associated virus. 

### 4.3. Berry Sampling

The field design of this project was a completely randomized design without blocking. Twenty and twenty-five data vines that tested positive (RB (+)) and negative (RB (−)) for GRBV were selected for each rootstock in 2016 and 2017, respectively. Data vines were further subdivided into four and five vines for each vineyard replicate in 2016 and 2017, respectively (*n* = 5). Vines were sampled every two weeks pre-*veraison* and weekly two weeks after *veraison* until harvest. Fifteen berries were randomly collected from different parts of the cluster and canopy of each vine and used to determine ripening progression. At harvest, the sampling was wider to include the vines utilized for winemaking. The values from the data vines regarding °Brix, pH, and TA (Table 1), were compared to the values of asymptomatic and symptomatic vines (Table S3), which agreed, indicating that symptomology is a strong indicator of virus status. Primary metabolites and components of harvest yield were measured from each data vines replicate (*n* = 5). For RB (+) and RB (−), 500 berries were randomly collected from harvest lots and stored at −80 °C until phenolic analysis and volatile aroma compound analysis could be performed. 

### 4.4. Grape Analysis through Ripening

Upon sampling, 25 berries for each replicate (five berries per data vine) were immediately processed. The juice from the 25 berries was collected and centrifuged at 3220× *g* at 4 °C for 15 min with an Eppendorf 5403 centrifuge (Westbury, NY, USA). Juice samples were then analyzed for total soluble solids (TSS) with a refractometer RFM110 (Bellingham + Stanley Ltd., UK), pH with an Orion-5-Star pH meter (Thermo Fisher Scientific Inc., Waltham, MA, USA) and titratable acidity (TA) with an DL50 Graphix titrator (Metter-Tolledo Inc., Columbus, OH, USA). The remaining berries were stored at −80 °C for future analysis. 

The skins were used to determine anthocyanin accumulation in the berries during ripening, since anthocyanins are localized in the pericarp of grape berries for non teinturier varieties [69]. From the berries stored at −80 °C, 15 berries from each biological replicate (three berries per data vine) at each collection date were accurately weighed, and the skins of the berries were removed using a scalpel. An acidified ethanol solution (1:1 ethanol:water, 0.1% ascorbic acid (*w/v*), and 0.1% HCl (*v/v*)) was added in ratio of 1:10 *w/v*, and the solution homogenized for three minutes 1355× *g* using an IKA ULTRA-TURRAX^®^T18 basic homogenizer (IKA^®^ Works, Inc., Wilmington, NC, USA). The solution extracted overnight for 18 h at 4°C and was then centrifuged at 3220× *g* at 4 °C for 15 min. The supernatant was collected, concentrated under reduced pressure at 34 °C, and quantitatively transferred to a 5 mL volumetric flask with acidified methanol. Anthocyanin concentration was then determined using a Genesys10S UV-Vis Spectrophotometer (Thermo Fisher Scientific, Madison, WI, USA) with similar protocols as in Harbertson et al. [70]. In summary, an aliquot of grape extract was diluted using model wine (0.5% sodium bitartrate *w/v* and 12% ethanol *v/v* adjusted to pH 3.3) to fit the absorbance limitations (0.1–1.2) of the spectrophotometer. Then, 100 µL of the diluted grape extract was added to a disposable cuvette along with 400 µL of model wine and 1 mL of an anthocyanin buffer (2.3% maleic acid (*w/v*) and 0.99% NaCl (*w/v*) adjusted to pH 1.8). Anthocyanins (expressed as malvidin-3-glucoside equivalents (M3G)) were measured at 520 nm and concentrations were calculated as in Harbertson et al. [70].

### 4.5. Grape Analysis at Harvest

#### 4.5.1. Grape Phenolic Profile

For the phenolic extraction, five sets of 20 berries from the RB (−) and RB (+) grapevines at harvest were randomly selected from grapes stored at −80 °C and weighed. Phenolic compounds were extracted similar to that described for anthocyanins (see Section 4.4) with the addition of a subsequent extraction with an acetone solution (70:30 acetone:water and 0.1% ascorbic acid (*w/v*)) in the same ratio of 1:10 *w/v*. After an 18-h, overnight extraction at 4 °C, the solution was centrifuged, and the supernatant collected. The ethanol and acetone extractions were combined, concentrated under reduced pressure at 34 °C, quantitatively transferred to a 10 mL volumetric flask with acidified methanol (1:1 methanol:water, 0.1% HCl (*v/v*)), and stored at −20 °C for up to one month until analysis was performed.

A modified protein precipitation assay was used to determine total phenolics, total anthocyanins, and total tannins [71]. Samples were thawed and diluted to fit the limitations of the spectrophotometer (0.1–1.2). Using a Genesys10S UV-Vis Spectrophotometer, total phenolics and total tannins were measured at 510 nm absorbance and expressed as catechin equivalents (CE); whereas total anthocyanins (expressed as M3G) were measured at 520 nm absorbance.

#### 4.5.2. Grape Volatile Profile

For the volatile extraction, five sets of 60 berries from the RB (−) and RB (+) grapevines collected at harvest were randomly selected from grapes stored at −80 °C and weighed. Samples were prepared similar to Hendrickson et al. [72] with a few adaptations. Briefly, 6 mL of a 0.83 M sodium citrate dihydrate solution (adjusted to pH of 6 with HCl) and 60 µL of a 200 g/L ascorbic acid solution was added to the grape berries. Each sample was spiked with 50 µL of a 10 mg/L 2-octanol internal standard solution. The grape berries were homogenized for one minute 1355× *g* using an IKA ULTRA-TURRAX^®^T18 basic homogenizer (IKA^®^ Works, Inc., Wilmington, NC, USA). The samples were then centrifuged at 3220× *g* at 4 °C for 15 min. Samples were analyzed in duplicate by transferring two-8 mL portions of supernatant to 20 mL amber headspace vials (Agilent Technologies, Santa Clara, CA, USA) containing 3 g of NaCl. Each vial was spiked with 50 µL of a 10 mg/L 2-undecanone internal standard solution. 

HS-SPME-GC-MS was used to analyze the volatile profiles of grape extracts. The instrument was controlled by a Gerstel Multi-Purpose Sampler (Maestro ver. 1.2.3.1 Gerstel). Headspace volatiles were extracted using a 100µm PDMS, Fused Silica Fiber (Supelco Analytical, Bellefonte, PA, USA). Samples were heated to 30 °C for five minutes under agitation, and then the PDMS fiber was introduced into the headspace of the sample vial and allowed to adsorb volatiles for 45 min. Once volatile adsorption was completed, the fiber was injected into the inlet which and volatiles desorbed at 260 °C for 10 min onto the column. Analysis was performed using an Agilent 7890A GC system equipped with a DB-WAXetr capillary column (30 m length × 250 µm internal diameter × 0.25 µm solid phase thickness) (Agilent Technologies, Santa Clara, CA, USA). The carrier gas, helium was kept at a constant pressure of 6.231 psi. The method was retention time locked to 2-undecanone and kept at a constant pressure to avoid retention time drift. The purge flow was 50 mL/min for 1.2 min, running on a splitless method. For GC analysis, the oven was kept at 40 °C for five minutes, then increased to 180 °C at 3 °C/min, and finally increased to 260 °C at 30 °C/min for a total run time of 60 min. The sample was transferred to a 5975C inert XL EI MSD with a triple-axis detector purchased from Agilent Technologies and ions were monitored using synchronous scan and selected ion monitoring (SIM). All compounds identified in this study were identified using the SIM mode as described in Hendrickson et al. [72]. Samples were analyzed using Mass Hunter software version B.07.00 (Agilent Technologies, Santa Clara, CA, USA). Compounds were semi-quantitatively analyzed using relative peak areas by normalization with 2-undecanone as well as the berry mass. Compounds were identified by retention time and confirmation of mass spectra ion peaks using the National Institute of Standards and Technology database (NIST) (https://www.nist.gov). Each grape sample replicate was analyzed in duplicate.

### 4.6. Weather Recordings

Precipitation, temperature, and growing degree days were collected from the University of California Agriculture and Natural Resources Integrated Pest Management Program (http://ipm.ucanr.edu/index.html) (Figure 1).

### 4.7. Statistical Analysis

Statistical analysis was conducted in the R language (R, version 3.6.1). All analyses used an α of 0.05 for statistical significances. One-way analysis of variance (ANOVA) and three-way ANOVA with three-way interactions were used to determine significant differences between samples. For a three-way ANOVA with three-way interactions, only the interactions of virus status to rootstock and virus status to year were considered to determine the influence genotypic or seasonal factors had on virus status. Virus status, rootstock, and year were all considered fixed effects for the purpose of determining the genotypic and temporal effects on disease status. A Tukey’s honestly significant difference (HSD) test was used for post hoc analysis. Principal component analysis (PCA) was used to display the variance in volatile analysis.

## 5. Conclusions

Geminiviruses threaten the productivity and quality of crops worldwide. GRBV is the first geminivirus to be detected in grapevines and our understanding of the detrimental impacts on grape and wine composition and quality is advancing. In this study CS on 420A rootstock was less sensitive to GRBV infection then CS on 110R rootstock. This was seen in anthocyanin and sugar accumulation in 2017, as well as the grape volatile profiles. This study also clearly indicated for the first time that the aroma profiles of grapes are also impacted by GRBV. We hypothesize that the difference in vigor and drought resistance in the two rootstocks led to a difference in microclimate of the grapevine and berry composition. Moreover, it was observed that seasonal differences considerably impact disease outcome in grapevines, mainly observed on primary metabolites such as sugars and organic acids. Further research into the transcriptome and metabolome of GRBV infected grapevines is needed to elucidate how these factors affect differential gene expression. In addition, these effects need to be evaluated in overall wine composition and quality.

## Figures and Tables

**Table 1:** °Brix, pH, TA (g/L), yield (kg) per vine, number of clusters per vine, and cluster mass (g) measurements from CS110R and CS420A data vines at harvest in 2016 and 2017 (*n* = 5).

Sample	Harvest Date	°Brix	pH	TA (g/L)	Yield (kg)	Clusters/Vine	Cluster Mass (g)
CS 110R RB (−)	9/20/16	25.4 ± 0.4 a	3.7 ± 0.10 a	3.1 ± 0.2 b	4.7 ± 0.6 a	36.9 ± 3.8 a	127.9 ± 6.0 a
CS 110R RB (+)	9/20/16	21.9 ± 1.0 b	3.5 ± 0.10 b	4.5 ± 0.6 a	5.5 ± 1.3 a	41.2 ± 6.74 a	130.6 ± 9.0 a
CS 420A RB (−)	9/20/16	25.6 ± 0.5 a	3.6 ± 0.0 a	3.2 ± 0.2 b	4.2 ± 0.8 a	32.5 ± 2.9 a	128.7 ± 12.6 a
CS 420A RB (+)	9/20/16	22.0 ± 0.5 b	3.34± 0.1 b	4.3 ± 0.4 a	4.9 ± 0.9 a	32.6 ± 3.6 a	142.2 ± 26.9 a
CS 110R RB (−)	9/26/17	24.6 ± 0.0 a	3.5 ± 0.0 a	4.1 ± 0.1 b	6.0 ± 0.7 b	54.79 ± 1.4 b	108.9 ± 10.7 a
CS 110R RB (+)	9/26/17	22.4 ± 0.0 b	3.5 ± 0.0 a	4.8 ± 0.1 a	7.1 ± 0.7 a	59.04 ± 3.1 a	120.2 ± 8.0 a
CS 420A RB (−)	10/6/17	25.1 ± 0.0 a	3.7 ± 0.0 a	3.6 ± 0.1 a	5.8 ± 1.3 a	50.60 ± 4.2 a	114.9 ± 21.3 a
CS 420A RB (+)	10/6/17	23.8 ± 0.0 b	3.7 ± 0.0 a	3.9 ± 0.1 a	6.2 ± 0.6 a	54.00 ± 3.1 a	113.9 ± 10.1 a
Significant Effects							
V		***	***	***	*	*	
Y				**	***	***	***
R		**	*	**		***	
V × Y		***	***	**			
V × R							
Y × R		*	***	**			
V × Y × R							

TA= Titratable Acidity, CS = Cabernet Sauvignon, RB = red blotch, (−) = negative, (+) = positive, V = virus status, Y = year, and R = rootstock. Difference in lettering indicates a significant difference between RB (−) and RB (+) for each rootstock after applying Tuckey’s HSD test (*p* < 0.05). Asterisks indicate a significant difference between RB (−) and RB (+) after a three-way ANOVA (* = *p* < 0.05, ** = *p* < 0.01, *** = *p* < 0.001).

## Supplementary Materials

The following are available online at https://www.mdpi.com/article/10.3390/plants10081583/s1, Table S1: Phenolic content (mg/berry) and concentrations (mg/g berry) of grape extracts at harvest determined through protein precipitation assay across rootstocks and seasons. Table S2: HS-SPME-GC-MS analysis of volatile compound content (mg/berry) in grapes at harvest (*n* = 5). Table S3: °Brix, pH, TA (g/L), YAN (mg/L), malic acid (mg/L) measurements from CS110R and CS420A symptomatic and asymptomatic vines used for winemaking in 2016 and 2017 (*n* = 3).
