# Supplementary material for: Impact of Rootstock and Season on Red Blotch Disease Expression in Cabernet Sauvignon (V. vinifera)
Source: Plants (Basel). 2021 Jul 31;10(8):1583. doi: 10.3390/plants10081583 (PMC8401632; doi:10.3390/plants10081583)
Supplement: Supplementary file 1 [file plants-10-01583-s001.zip › plants-1312194-supplementary.pdf]

Table S1. Phenolic content (mg/berry) and concentrations (mg/ g berry) of grape extracts at harvest determined through protein precipitation assay across rootstocks and seasons. The main effects, two-way, and three-way interactions ANOVA were determined for content and concentration for each class of compounds.

mg/berry				mg/g berry			
**2016**	**Total Phenolics**	**Total Anthocyanins**	**Total Tannins**	**2016**	**Total Phenolics**	**Total Anthocyanins**	**Total Tannins**
**CS 110R RB (−)**	7.54 ± 0.41	1.57 ± 0.43	3.04 ± 0.21	**CS 110R RB (−)**	6.54 ± 0.17	1.28 ± 0.40	2.64 ± 0.15
**CS 110R RB (+)**	8.60 ± 0.27	1.54 ± 0.16	3.31 ± 0.13	**CS 110R RB (+)**	7.55 ± 0.20	1.31 ± 0.17	2.90 ± 0.069
**CS 420A RB (−)**	9.49 ± 0.68	0.76 ± 0.079	3.82 ± 0.19	**CS 420A RB (−)**	7.42 ± 0.52	0.66 ± 0.024	2.99 ± 0.15
**CS 420A RB (+)**	10.25 ± 0.77	0.68 ± 0.12	3.47 ± 0.33	**CS 420A RB (+)**	6.94 ± 0.77	0.60 ± 0.07	2.35 ± 0.32
**2017**	**Total Phenolics**	**Total Anthocyanins**	**Total Tannins**	**2017**	**Total Phenolics**	**Total Anthocyanins**	**Total Tannins**
**CS 110R RB (−)**	8.22 ± 0.96	1.30 ± 0.18	6.24 ± 0.79	**CS 110R RB (−)**	8.73 ± 0.61	1.37 ± 0.14	6.62 ± 0.47
**CS 110R RB (+)**	9.72 ± 1.46	0.84 ± 0.14	7.23 ± 1.04	**CS 110R RB (+)**	11.72 ± 0.83	0.85 ± 0.10	8.73 ± 0.65
**CS 420A RB (−)**	11.22 ± 1.09	2.63 ± 0.35	9.01 ± 0.84	**CS 420A RB (−)**	10.85 ± 0.68	2.81 ± 0.31	8.71 ± 0.42
**CS 420A RB (+)**	9.78 ± 0.43	2.33 ± 0.20	7.77 ± 0.37	**CS 420A RB (+)**	9.65 ± 0.43	2.44 ± 0.20	7.66 ± 0.31
**Significant Effects**				**Significant Effects**			
**V**		**		**V**	***	**	*
**Y**	**	***	***	**Y**	***	***	***
**R**	***	***	***	**R**	*	***	**
**V x Y**		*		**V x Y**		**	*
**V x R**	**		***	**V x R**	***		***
**Y x R**		***	**	**Y x R**	*	***	
**V x Y x R**	*		*	**V x Y x R**	***		***

CS = Cabernet Sauvignon, RB = red blotch, (−) = negative, (+) = positive, V = virus status, Y = year, and R = rootstock. Asterisks indicate a significant difference between RB (−) and RB (+) after an ANOVA (* = $p < 0.05$, ** = $p < 0.01$, *** = $p < 0.001$).

Table S2. HS-SPME-GC-MS analysis of volatile compound content (mg/berry) in grapes at harvest (n = 5).

Compound (mg/berry)	2017				2016			
	CS 110R RB (−)	CS 110R RB (+)	CS 420A RB (−)	CS 420A RB (+)	CS 110R RB (−)	CS 110R RB (+)	CS 420A RB (−)	CS 420A RB (+)
**Ethyl Acetate †Φ**	0.92 ± 0.45	3.88 ± 1.22	0.07 ± 0.03	0.07 ± 0.05	2.27 ± 0.49	0.59 ± 0.17	1.32 ± 0.37	1.00 ± 0.29
**Hexanal**	4.73 ± 1.12	1.55 ± 0.99	3.94 ± 2.35	4.84 ± 2.91	0.10 ± 0.02	0.15 ± 0.04	0.11 ± 0.02	0.17 ± 0.04
**β-Myrcene**	0.03 ± 0.02	0.01 ± 0.00	0.03 ± 0.01	0.03 ± 0.02	0.49 ± 0.11	0.35 ± 0.08	0.40 ± 0.09	0.30 ± 0.09
**Limonene #Φ**	0.12 ± 0.05	0.07 ± 0.03	0.12 ± 0.02	0.14 ± 0.05	0.29 ± 0.03	0.20 ± 0.03	0.22 ± 0.04	0.20 ± 0.04
**2-Hexenal †#Φ**	1.61 ± 0.61	0.82 ± 0.39	1.98 ± 0.74	3.04 ± 1.79	7.14 ± 1.50	13.33 ± 3.09	10.78 ± 1.37	15.97 ± 2.09
**Ehtyl Hexanoate †#Φ**	0.18 ± 0.09	0.21 ± 0.12	0.06 ± 0.02	0.04 ± 0.04	0.49 ± 0.07	0.26 ± 0.08	0.32 ± 0.08	0.30 ± 0.11
**p-Cymene #Φ**	0.01 ± 0.00	0.01 ± 0.00	0.01 ± 0.00	0.01 ± 0.00	0.04 ± 0.00	0.02 ± 0.00	0.02 ± 0.00	0.02 ± 0.00
**Hexyl acetate †Φ**	0.04 ± 0.03	0.07 ± 0.03	0.03 ± 0.00	0.03 ± 0.01	0.61 ± 0.08	0.38 ± 0.07	0.46 ± 0.07	0.40 ± 0.06
**Octanal Φ**	0.02 ± 0.01	0.01 ± 0.01	0.02 ± 0.00	0.03 ± 0.01	0.13 ± 0.02	0.13 ± 0.03	0.18 ± 0.04	0.18 ± 0.05
**Hexanol †**	1.72 ± 0.43	1.62 ± 0.60	1.52 ± 0.29	1.91 ± 1.23	10.36 ± 2.31	9.22 ± 3.40	8.19 ± 1.30	9.66 ± 1.88
**trans-3-Hexen-1-ol †**	0.11 ± 0.04	0.09 ± 0.03	0.11 ± 0.02	0.14 ± 0.09	1.09 ± 0.25	0.73 ± 0.20	0.78 ± 0.18	0.78 ± 0.15
**cis-3-Hexen-1-ol †#**	0.28 ± 0.09	0.39 ± 0.14	0.25 ± 0.06	0.59 ± 0.30	1.18 ± 0.35	2.97 ± 1.19	1.33 ± 0.19	2.86 ± 0.54
**trans-2-Hexen-1-ol †#Φ**	6.11 ± 2.24	5.98 ± 2.20	6.02 ± 1.02	7.98 ± 4.96	7.03 ± 1.58	6.03 ± 2.42	5.38 ± 0.86	7.63 ± 1.27
**Ethyl Octanoate**	0.02 ± 0.02	0.01 ± 0.01	0.95 ± 0.05	1.36 ± 0.58	0.05 ± 0.01	0.03 ± 0.01	1.98 ± 0.35	2.42 ± 0.33
**Nerol oxide #**	0.02 ± 0.03	0.01 ± 0.01	0.09 ± 0.03	0.12 ± 0.07	0.17 ± 0.02	0.14 ± 0.02	0.43 ± 0.10	0.37 ± 0.16
**Benzaldehyde #**	0.09 ± 0.03	0.06 ± 0.02	0.003 ± 0.00	0.01 ± 0.00	0.61 ± 0.14	0.40 ± 0.10	0.14 ± 0.02	0.15 ± 0.02
**β-linalool**	0.01 ± 0.00	0.01 ± 0.00	0.05 ± 0.01	0.06 ± 0.03	0.08 ± 0.02	0.05 ± 0.01	0.42 ± 0.07	0.46 ± 0.10
**Geranial †#**	0.02 ± 0.00	0.02 ± 0.01	0.03 ± 0.01	0.04 ± 0.01	0.07 ± 0.01	0.04 ± 0.00	3.02 ± 0.13	3.02 ± 0.29
**β-Damascenone †**	0.01 ± 0.00	0.02 ± 0.01	0.01 ± 0.00	0.01 ± 0.00	0.03 ± 0.01	0.02 ± 0.01	0.05 ± 0.01	0.04 ± 0.01
**Benzyl alcohol #**	0.19 ± 0.049	0.12 ± 0.05	0.004 ± 0.00	0.01 ± 0.00	0.79 ± 0.15	0.52 ± 0.14	0.02 ± 0.01	0.03 ± 0.01
**2-Phenethyl alcohol**	0.22 ± 0.05	0.15 ± 0.07	0.14 ± 0.05	0.15 ± 0.06	1.14 ± 0.22	0.74 ± 0.15	0.29 ± 0.07	0.22 ± 0.07
**β-Ionone Φ**	0.01 ± 0.00	0.01 ± 0.01	0.11 ± 0.03	0.11 ± 0.06	0.03 ± 0.00	0.01 ± 0.00	0.45 ± 0.09	0.46 ± 0.10
**Ethyl cinnamate †**	0.01 ± 0.01	0.02 ± 0.00	0.01 ± 0.00	0.01 ± 0.00	0.07 ± 0.04	0.03 ± 0.01	0.02 ± 0.00	0.02 ± 0.00

CS = Cabernet Sauvignon, RB = red blotch, (−) = negative, (+) = positive, † = volatile compound has significant virus status to year effect, # = volatile compound has significant virus status to rootstock effect, Φ = volatile compound has significant virus status effect.

Table S3. °Brix, pH, TA (g/L), YAN (mg/L), malic acid (mg/L) measurements from CS110R and CS420A symptomatic and asymptomatic vines used for winemaking in 2016 and 2017 (n=3).

Sample	Harvest Date	°Brix	pH	TA (g/L)	Malic Acid (mg/L)
**CS 110R RB (−)**	**9/20/16**	25.6 ± 0.1 a	3.6 ± 0.0 a	3.8 ± 0.3 b	1460.0 ± 55. b
**CS 110R RB (+)**	**9/20/16**	21.7 ± 0.1 b	3.5 ± 0.0 a	4.8 ± 0.1 a	2275.0 ± 48.6 a
**CS 420A RB (−)**	**9/20/16**	24.3 ± 0.1 a	3.5 ± 0.0 a	4.2 ± 0.1 b	1625.7 ± 48.0 b
**CS 420A RB (+)**	**9/20/16**	22.1 ± 0.1 b	3.5 ± 0.0 a	4.5 ± 0.1 a	1852.0 ± 13.9 a
**CS 110R RB (−)**	**9/26/17**	25.5 ± 0.1 a	3.6 ± 0.0 a	4.0 ± 0.0 b	2649.3 ± 45.7 a
**CS 110R RB (+)**	**9/26/17**	23.4 ± 0.0 b	3.6 ± 0.0 a	4.9 ± 0.1 a	2779.0 ± 68.6 a
**CS 420A RB (−)**	**10/6/17**	25.3 ± 0.1 a	3.6 ± 0.0 a	4.6 ± 0.1 a	2201.0 ± 34.7 b
**CS 420A RB (+)**	**10/6/17**	23.6 ± 0.3 b	3.5 ± 0.0 a	4.8 ± 0.0 a	2870.0 ± 21.0 a

TA = Titratable Acidity, CS110 = CS 110R, CS420 = CS 420A, RB = red blotch, (−) = negative, and (+) = positive. Difference in lettering indicates a significant difference between RB (−) and RB (+) after applying Tuckey's HSD test ($p < 0.05$).
